# The Risks of Clinical Expression of Alzheimer’s Disease (AD) in People with Biomarkers of Alzheimer's Pathology

**DOI:** 10.1002/alz70859_107831

**Published:** 2025-12-25

**Authors:** Howard H. Feldman, Nicolas Villain, Giovanni B. Frisoni, Bruno Dubois

**Affiliations:** ^1^ Alzheimer's Disease Cooperative Study, University of California San Diego, La Jolla, CA USA; ^2^ Department of Neurosciences, University of California, San Diego, La Jolla, CA USA; ^3^ Département de Neurologie, Groupe Hospitalier Pitié‐Salpêtrière, AP‐HP Sorbonne Université, Institute of Memory and Alzheimer’s Disease, Paris France; ^4^ Sorbonne Université, INSERM U1127, CNRS 7225, Institut du Cerveau ‐ ICM, Maladie d’Alzheimer, Maladies à Prions, Paris, France, Paris France; ^5^ Laboratory of Neuroimaging of Aging, University of Geneva, Geneva Switzerland; ^6^ Memory Clinic, University Hospital of Geneva, Geneva Switzerland; ^7^ Sorbonne Université, INSERM U1127, CNRS 7225, Institut du Cerveau ‐ ICM, FrontLab, Paris France

## Abstract

**Background:**

The widespread availability of biomarkers of Alzheimer’s pathology (BAP) opens new diagnostic considerations for those who have not clinically expressed the disease but who are identified as being at increased risk based on their BAP. The International Working Group (IWG) updated their recommendations within a patient‐centered nosographic approach that considers clinical evaluation as a cornerstone with attention to risk and resilience factors, patterns of biomarkers, comorbidities, genetics, and other test results (Dubois B, et al. JAMA Neurol 2024). They stratify risk and tie it to a specific clinical patient journey, including the communication of risk, tailored management of modifiable risk factors, counselling, multidomain lifestyle interventions, and treatment research considerations.

**Method:**

For these updated IWG recommendations, an evidence review of available literature between July 1, 2020 and March 2024 was undertaken with a variety of biomarker search terms and AD. The search included a review of papers focused on near term and lifetime risks of progression to AD dementia in cognitively unimpaired people with different BAP patterns.

**Result:**

These IWG recommendations provide a risk stratification framework for unimpaired individuals with classification into “Asymptomatic At‐Risk” and “Presymptomatic”. The majority of cognitively unimpaired individuals with BAP, including those with an amyloid or AD pathophysiological biomarker, will not express clinical AD in their lifetime. The Presymptomatic group includes those with fully penetrant monogenic mutations, Down syndrome, or alternatively patterns of positive amyloid biomarker with neocortical tau PET biomarkers that reach the threshold of near certainty of clinical expression of AD. Prioritizing the testing of preventive disease modifying treatments is identified as urgent for this Presymptomatic group. Furthermore, it is expected that this group will expand as new evidence and patterns of biomarkers reach the necessary thresholds for their inclusion. Management plans can be further tailored to this classification framework.

**Conclusion:**

This IWG risk stratification classification recommends against diagnosis of AD based on BAP alone without its clinical expression. This AD classification lends itself to a tailored management approach and specific patient journeys for each of its groups.

